# The Structure–Function Link of Ellipsoid Zone Integrity and Outer Retinal Features with Visual Acuity in Dry AMD

**DOI:** 10.3390/diagnostics16182925

**Published:** 2026-09-10

**Authors:** Reem Amine, Masaharu Mizuno, Karen Matar, Asmita Indurkar, Paulo Simoes, Ming Hu, Rachel A. Downes, Katherine E. Talcott, Jamie L. Reese, Justis P. Ehlers

**Affiliations:** 1 The Tony and Leona Campane Center for Excellence in Image-Guided Surgery and Advanced Imaging Research, Cleveland Clinic, Cleveland, OH 44195, USA; reem.amine8@gmail.com (R.A.); mizunom@ccf.org (M.M.);; 2 Cole Eye Institute, Cleveland Clinic, Cleveland, OH 44195, USA; 3 Department of Genomic Sciences and Systems Biology, Cleveland Clinic Research, Cleveland Clinic Foundation, Cleveland, OH 44195, USA

**Keywords:** age-related macular degeneration, geographic atrophy, ellipsoid zone integrity, biomarkers, visual acuity

## Abstract

**Background/Objectives:** The purpose of this study was to evaluate associations between quantitative ellipsoid zone (EZ) integrity metrics derived from spectral-domain OCT (SD-OCT) and visual outcomes, including clinically meaningful visual acuity (VA) loss, in dry age-related macular degeneration (AMD). **Methods:** This single-center retrospective image-analysis cohort included 351 eyes from 351 patients with intermediate AMD or atrophic changes. Macular SD-OCT scans were analyzed using a certified-reader-validated machine learning-based segmentation platform to delineate the EZ, retinal pigment epithelium (RPE), and Bruch’s membrane (BM). EZ metrics included EZ-RPE thickness (i.e., outer segment thickness) and EZ attenuation, defined as partial attenuation (EZ-RPE thickness of ≤20 µm, abnormal outer segment thinning)) or total attenuation/loss (0 µm). Drusen volume was quantified from the RPE-BM compartment. VA was converted to ETDRS letters. Associations between baseline OCT metrics and baseline VA, Year 2 VA, and ≥15-letter VA loss were assessed using nonparametric testing, linear regression, and ANCOVA. **Results:** Greater EZ-RPE thickness and lower EZ attenuation were associated with better baseline VA (all *p* < 0.001), and EZ metrics remained significant in cross-sectional regression models. Among 188 eyes with Year 2 follow-up, 38 (20%) experienced ≥15-letter VA loss. These eyes had significantly lower baseline EZ-RPE thickness (i.e., abnormal outer segment thinning) and greater EZ attenuation than eyes without such loss (all *p* < 0.001). In ANCOVA models, EZ metrics remained associated with Year 2 VA (all *p* < 0.001), whereas drusen volume was not. **Conclusions:** Quantitative EZ integrity metrics were associated with cross-sectional and longitudinal visual outcomes in dry AMD. EZ-RPE thickness and EZ attenuation may serve as clinically relevant OCT biomarkers reflecting photoreceptor integrity and risk of meaningful VA loss.

## 1. Introduction

Age-related macular degeneration (AMD) is the leading cause of irreversible central vision loss among older adults worldwide, affecting nearly 200 million people, a number that is projected to increase substantially with global population aging [1]. AMD is clinically classified into early, intermediate (iAMD), and advanced stages [2,3,4]. Advanced AMD includes either geographic atrophy (GA) or neovascular AMD (nAMD), both associated with significant visual loss. GA, a late-stage form of dry AMD, affects approximately 15–20% of individuals aged ≥85 years with affected individuals experiencing a profound functional decline and reduced quality of life [5]. Although intravitreal complement inhibitors have recently been approved and shown to modestly reduce the rate of GA enlargement [6,7], no available therapy can restore lost photoreceptors or reverse established atrophy. This underscores the need for reliable biomarkers that can identify high-risk patients, inform prognosis, and serve as sensitive surrogate endpoints in clinical trials [8]. Optical coherence tomography (OCT) has transformed the evaluation of AMD by enabling high-resolution visualization of the outer retina [9]. Among OCT-derived features, the ellipsoid zone (EZ), the mitochondria-rich portion of photoreceptor inner segments, has emerged as a critical marker of photoreceptor integrity, with its preservation correlating closely with visual function [10]. Prior studies have shown that EZ disruption is associated with reduced retinal sensitivity on microperimetry and often precedes the development of complete retinal pigment epithelium and outer retinal atrophy (cRORA) [11]. Quantitative OCT assessment of EZ integrity encompasses complementary biomarkers, including EZ-RPE central thickness (i.e., a measure of outer segment thickness), EZ-RPE volume, and partial and total EZ attenuation, which provide objective and reproducible indicators of photoreceptor health [12].

Several investigations have examined the association between EZ integrity and vision in dry AMD. Pfau et al. demonstrated that EZ loss correlates strongly with reduced retinal sensitivity, supporting its role as an early structural biomarker [13]. In our prior work, we showed that central EZ thickness and attenuation were significantly associated with both baseline and future VA in a longitudinal cohort of eyes with dry AMD [14]. Collectively, these findings support EZ integrity as a structural correlate of visual function, analogous to how central retinal thickness is used as an outcome measure in nAMD [15,16]. Despite these important observations, several clinically relevant questions remain unresolved. Previous studies primarily evaluated individual EZ metrics, and the relative performance of different quantitative EZ biomarkers has not been systematically compared. In addition, the independent contribution of EZ integrity beyond other structural OCT biomarkers, including drusen volume and outer retinal layer (ONL)-RPE thickness, remains incompletely characterized. Importantly, the relationship between EZ metrics and the U.S. Food and Drug Administration (FDA)-recognized endpoint of ≥15-letter loss [17], a clinically meaningful decline equivalent to three lines, has not been fully established.

The present study addresses these gaps by systematically evaluating multiple quantitative EZ integrity metrics in a cohort of eyes with iAMD and atrophic changes imaged with standardized spectral-domain OCT (SD-OCT). We evaluated the relative associations of these biomarkers with both cross-sectional and longitudinal visual outcomes using multivariable analyses and assessed their relationship with the FDA-recognized endpoint of ≥15-letter visual acuity loss at Year 2.

## 2. Materials and Methods

### 2.1. Study Design and Population

This was a single-center, retrospective image analysis study of patients imaged at the Cleveland Clinic Cole Eye Institute (Cleveland, OH, USA). This study was IRB-approved (14-1527) and adhered to the tenets of the Declaration of Helsinki. The requirement for informed consent was waived by the IRB due to the retrospective nature of this study.

Eligible participants were aged 55 years or older and had dry AMD confirmed at baseline in the study eye, including iAMD and OCT-defined atrophy. Potential cases were identified through review of clinical documentation and the diagnosis was confirmed by certified graders based on baseline SD-OCT imaging. For eyes classified as iAMD, the diagnosis required the presence of large drusen on SD-OCT, defined as drusen with a minimum horizontal width of ≥125 µm. Detailed OCT-based definitions of atrophy and imaging metrics are provided below. Eyes were excluded if there was any history or evidence of nAMD, presence of intraretinal or subretinal fluid at baseline or during follow-up, prior anti-vascular endothelial growth factor (VEGF) therapy, concurrent retinal disease (e.g., diabetic macular edema, retinal vein occlusion, or pathologic myopia), prior vitreoretinal surgery, or poor-quality OCT scans precluding reliable segmentation of retinal layers. For patients meeting criteria in both eyes, the right eye was chosen. If only one eye met the eligibility criteria, that eye was included. For longitudinal analyses, eligible eyes were required to have follow-up evaluation at Year 2 (24 months ± 1 month). Eyes were excluded if they underwent cataract surgery during the follow-up period or if Year 2 visual acuity (VA) data were unavailable.

### 2.2. OCT Imaging and Analysis

SD-OCT imaging was performed using Cirrus HD-OCT 5000 (Carl Zeiss Meditec, Dublin, CA, USA; 512 × 128 macular cube, 6 × 6 mm). An automated machine learning-based multilayer segmentation platform was applied to delineate the ONL, EZ, RPE, and Bruch’s membrane (BM). All automated segmentations were reviewed by certified graders at the B-scan level while masked to all clinical information and study outcomes, and segmentation errors were manually corrected when necessary. The segmentation platform and grading workflow have been previously validated, demonstrating excellent inter-grader agreement and consistency for EZ-RPE thickness in dry AMD (ICC 0.967–0.988) [18,19]. For longitudinal analyses, Year 2 SD-OCT images were analyzed using the same segmentation pipeline and grading workflow as the baseline images.

### 2.3. OCT Metrics

**Figure 1:**
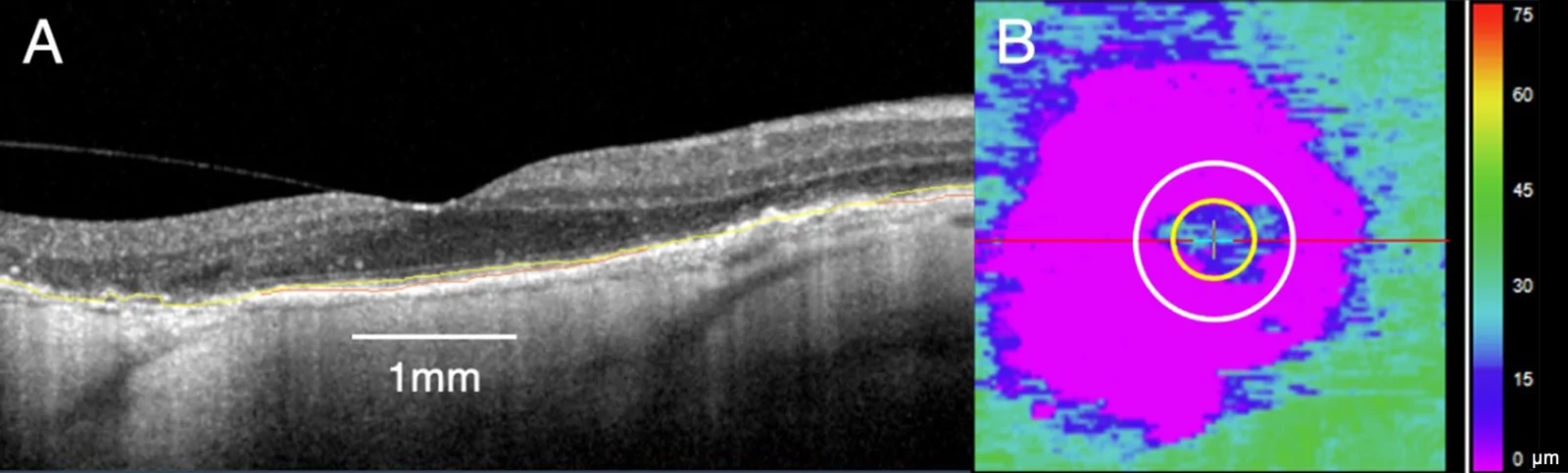
Representative OCT imaging of EZ integrity assessment. (**A**) SD-OCT B-scan illustrating the central 1 mm subfield (white bar). Segmentation boundaries of the EZ (yellow line) and RPE (red line) are highlighted. In regions where the EZ is not visible, the EZ segmentation is dropped to the RPE band, resulting in an effective EZ-RPE thickness of 0 μm and representing total EZ loss. Consequently, the EZ and RPE segmentation boundaries overlap and are displayed as a single yellow line for visualization purposes. (**B**) EZ integrity map derived from a 6 × 6 mm macular cube scan. The yellow circle corresponds to the central subfield (1 mm), and the white circle denotes the central macular (2 mm) region. EZ = ellipsoid zone; SD-OCT = spectral-domain optical coherence tomography; RPE = retinal pigment epithelium.

Quantitative OCT metrics were derived from the automated segmentation pipeline and included EZ-RPE central thickness, ONL-RPE thickness, EZ-RPE volume, and EZ attenuation. EZ attenuation was defined as the percentage of the analyzed region exhibiting EZ-RPE thickness ≤ 20 µm (partial attenuation) or EZ-RPE thickness = 0 µm (total attenuation), based on previously described OCT-based definitions of EZ structural attenuation [12,14]. Drusen volume was quantified from the RPE-BM compartment. OCT-defined atrophy was defined as a cumulative area of total RPE loss (RPE-BM thickness = 0 µm) derived from reviewed and manually corrected RPE and BM segmentations, measuring at least 0.05 mm^2^, corresponding to a minimum lesion diameter of 250 μm. This threshold was selected to be consistent with the minimum lesion size defined for cRORA [11], while serving as a quantitative OCT-based surrogate for atrophy rather than the traditional fundus photo-based criteria. This approach has been previously validated as an OCT-based surrogate that closely corresponds to FAF-defined geographic atrophy [20]. Metrics were quantified within predefined retinal regions according to metric-specific definitions, including the central subfield (1 -mm diameter), central macular region (2-mm diameter), and the panmacular region corresponding to the full 6 × 6 mm macular cube centered on the fovea, as applicable. The foveal center was manually identified on each scan, and all regional metrics were calculated relative to this manually defined center. Eyes with substantial scan decentration or inadequate foveal visualization were excluded from analysis. Central subfield atrophy status was determined at baseline based on the OCT-defined atrophy criteria and was used for stratified analyses. Representative examples of EZ integrity assessment are shown in Figure 1.

### 2.4. Visual Acuity Assessment

VA was obtained from medical records and converted from Snellen to logMAR and subsequently expressed in Early Treatment Diabetic Retinopathy Study (ETDRS) letters for analysis. Importantly, due to the retrospective nature of this study, VA represented the best recorded vision in the chart (i.e., based on pinhole or refraction). No standardized refractions were performed. For cross-sectional analyses, VA was categorized into three clinically relevant strata based on ETDRS letter score: ≥80 letters (approximately Snellen 20/25 or better), >70 to <80 letters, and ≤70 letters (20/40 or worse). For longitudinal analyses, clinically meaningful VA loss was defined as a decrease of ≥15 ETDRS letters from baseline to the Year 2 visit.

### 2.5. Outcomes

Primary outcomes included the associations between baseline EZ integrity metrics and baseline VA (cross-sectional analyses), as well as Year 2 VA, and ≥15-letter VA loss at Year 2 (longitudinal analyses).

### 2.6. Statistical Analysis

Continuous variables were summarized as median (IQR) given non-normal distributions based on the Shapiro–Wilk test. Group comparisons were performed with Mann–Whitney U or Kruskal–Wallis tests, and categorical variables with χ^2^ tests. Associations were evaluated with Spearman correlation. Multivariable linear regression analyses were performed to evaluate associations between baseline VA and EZ integrity metrics, with age and sex included as covariates. EZ metrics and drusen volume were entered simultaneously, and additional analyses were stratified by baseline central subfield atrophy status. Drusen volume was rescaled and entered per 0.1 mm^3^ to improve interpretability of regression coefficients. To assess potential selection bias, baseline demographic, functional, and structural characteristics were compared between eyes included in and excluded from the longitudinal cohort. For longitudinal outcomes, multivariable logistic regression models adjusted for age and sex were used to evaluate associations between baseline OCT metrics and ≥15-letter VA loss at Year 2. Model discrimination was assessed using ROC curve analysis and AUC values. ANCOVA models were used to evaluate associations between baseline EZ metrics and Year 2 VA, adjusting for baseline VA, age, sex, and drusen volume. Because EZ integrity metrics are highly correlated measures of photoreceptor structure, they were evaluated in separate multivariable models to avoid multicollinearity. Additional multivariable models incorporating ONL-RPE thickness were performed to evaluate its association with visual outcomes. A two-sided *p* < 0.05 was considered statistically significant. All analyses were performed using SPSS Statistics, version 29.0.1.0 (IBM, Armonk, NY, USA) and R software, version 4.5.1 (R Foundation for Statistical Computing, Vienna, Austria).

## 3. Results

### 3.1. Study Population

A total of 351 eyes from 351 patients met the inclusion criteria. The median age was 80 years, and 58% of patients were female. At baseline, the median VA was 76 ETDRS letters (approximately 20/32 Snellen equivalent), and atrophy was present in 36% of eyes. Baseline demographic and clinical characteristics are summarized in Table 1.

### 3.2. Cross-Sectional Associations with Baseline VA

**Figure 2:**
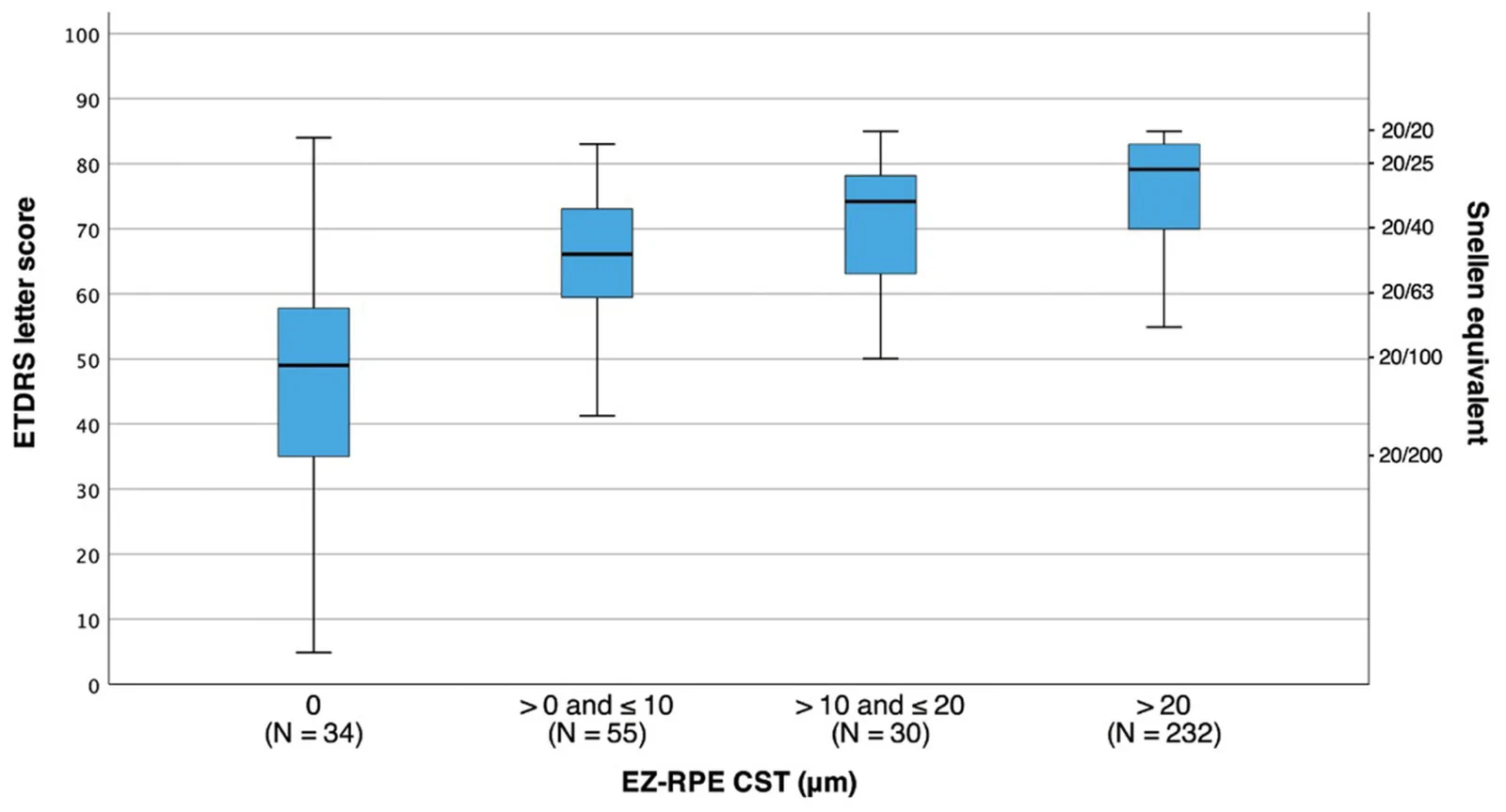
Baseline ETDRS letter scores stratified by EZ-RPE thickness categories. Boxplots display the median and interquartile range, with whiskers indicating the range. Sample sizes for each group are shown below the *x*-axis. ETDRS = Early Treatment Diabetic Retinopathy Study; EZ = ellipsoid zone; RPE = retinal pigment epithelium.

When stratified by baseline VA categories (≥80, >70 to <80, ≤70 ETDRS letter, corresponding approximately to ≥20/25 and ≤20/40 Snellen acuity), the eyes with better VA consistently demonstrated greater EZ integrity across all evaluated metrics. EZ-RPE central thickness progressively decreased with worsening VA, while total EZ attenuation increased substantially, with the eyes in the ≤70 letters showing a median attenuation of 36.7% in the central subfield (*p* < 0.001; Table 2). Consistent with this analysis, Figure 2 visualizes baseline VA distributions across EZ-RPE central subfield thickness (CST) strata, showing lower VA values and greater dispersion in visual outcomes in lower CST strata. Similar monotonic patterns were observed for partial EZ attenuation, EZ-RPE volume, and ONL-RPE thickness, with progressively greater attenuation and thinner outer retinal layers in the eyes with worse baseline VA. Spearman correlation analyses using baseline VA as a continuous outcome demonstrated significant positive correlations between VA and EZ-RPE central thickness, EZ-RPE volume, and ONL-RPE thickness, and significant negative correlations with EZ attenuation metrics (all *p* < 0.001; Table 2).

### 3.3. Cross-Sectional Multivariable Analysis of EZ Integrity and Drusen Volume

In the overall cohort, EZ-RPE CST, partial EZ attenuation, and total EZ attenuation were all significantly associated with baseline VA in multivariable models. Drusen volume was also significantly associated with VA in most models, depending on the EZ metric included (Table 3). When the analyses were stratified by central subfield atrophy status, EZ integrity metrics remained significantly associated with baseline VA in both the eyes without central subfield atrophy and the eyes with central subfield atrophy present. In contrast, drusen volume was not significantly associated with baseline VA in either stratified subgroup across models (Table 3).

### 3.4. Longitudinal Associations with Year 2 VA Outcomes

Of the 351 eyes included in the cross-sectional cohort, 188 eyes were eligible for longitudinal analysis after excluding eyes that underwent cataract surgery during follow-up or eyes lacking Year 2 VA data. Baseline demographic, functional, and structural characteristics did not differ significantly between the eyes included in and those excluded from the longitudinal analysis (Table S1). At Year 2, 38 eyes (20%) experienced ≥15-letter loss. Baseline EZ integrity differed between the eyes with stable VA and those with significant VA loss. The eyes that experienced ≥15-letter loss had lower baseline EZ-RPE central thickness and greater partial and total EZ attenuation across both the central subfield and central macular regions (all *p* < 0.001; Table 4). In contrast, baseline drusen volume did not differ significantly between groups. Spearman correlation analyses using Year 2 VA as a continuous outcome demonstrated significant correlations between baseline EZ integrity metrics and visual outcomes at Year 2, with greater EZ-RPE central thickness associated with better VA and greater EZ attenuation associated with worse VA (Table 4). Longitudinal changes in EZ integrity metrics were quantified from paired baseline and Year 2 OCT images using the same segmentation pipeline. The changes in EZ-RPE thickness and partial and total EZ attenuation, stratified by ≥15-letter VA loss status, are summarized in Table S2.

### 3.5. Multivariable Models for Year 2 Visual Outcomes

The base logistic regression model including age and sex demonstrated modest discrimination for ≥15-letter loss (M1; AUC = 0.66). The addition of EZ-RPE CST (M2; AUC = 0.76), partial EZ attenuation (M3; AUC = 0.77), or total EZ attenuation (M4; AUC = 0.73) to the base model improved model discrimination. All EZ metrics remained independently associated with ≥15-letter loss (Table 5, Panel A). In contrast, the ANCOVA models adjustedaddition of ONL-RPE thickness (M5; AUC = 0.69) or drusen volume (M6; AUC = 0.66) did not improve model discrimination relative to the base model and were not significantly associated with ≥15-letter loss (Table 5, Panel A). In the ANCOVA models adjusted for baseline VA, age, sex, and drusen volume, all EZ metrics were significantly associated with Year 2 VA. EZ-RPE CST was positively associated with Year 2 VA, whereas both partial and total EZ attenuation were negatively associated with visual outcomes (all *p* < 0.001). In contrast, drusen volume was not significantly associated with Year 2 VA across models (Table 5, Panel B).

### 3.6. Models Including ONL-RPE Thickness

In cross-sectional linear regression models for baseline VA, both total EZ attenuation and ONL-RPE thickness were significantly associated with VA (both *p* < 0.001; Table S3, Panel A). In contrast, in ANCOVA models evaluating Year 2 VA, total EZ attenuation remained significantly associated with visual outcomes (*p* < 0.001), whereas ONL-RPE thickness was not associated with Year 2 VA (*p* = 0.455; Table S3, Panel B).

## 4. Discussion

This study demonstrated that quantitative EZ integrity metrics were associated with both baseline VA and the risk of future VA decline in eyes with dry AMD. Across cross-sectional and longitudinal analyses, greater EZ attenuation and reduced EZ-RPE central thickness and volume were consistently linked with worse VA. Importantly, in the longitudinal cohort, worse baseline EZ integrity was associated with the clinically meaningful endpoint of ≥15-letter loss at Year 2, underscoring their potential value as structural biomarkers associated with clinically meaningful visual outcomes.

The present findings further suggest that the EZ integrity features most strongly associated with visual function differ depending on the temporal context of the outcome. For baseline VA, metrics reflecting established central photoreceptor loss, particularly central subfield total EZ attenuation, showed the strongest associations, consistent with their direct correspondence to current foveal structural damage. In contrast, for visual outcomes assessed at Year 2, measures capturing residual foveal structure and partially compromised photoreceptor regions, including EZ-RPE CST and central subfield partial EZ attenuation, demonstrated stronger relationships with subsequent visual acuity. This pattern is biologically plausible. Central subfield total EZ attenuation represents advanced structural disruption and therefore closely reflects contemporaneous visual function. Conversely, preserved EZ-RPE CST and regions of central subfield partial EZ attenuation may represent structurally vulnerable but still functional photoreceptor populations that convey prognostic information regarding future visual decline. Accordingly, central subfield total EZ attenuation appears most informative for characterizing current visual status, whereas EZ-RPE CST and central subfield partial EZ attenuation may better capture at-risk foveal architecture relevant to longitudinal outcomes.

The present study also highlights the importance of spatial scale when interpreting EZ integrity metrics in relation to visual function. Because VA primarily reflects foveal cone-mediated vision, it is not surprising that EZ metrics quantified within the central subfield demonstrated the strongest associations with both baseline and longitudinal visual outcomes. In particular, EZ-RPE CST and central subfield EZ attenuation were most closely linked to VA, underscoring the central role of foveal photoreceptor integrity in determining visual acuity. EZ integrity metrics assessed within the central macular region (2 mm) and across the full 6 × 6 mm macular cube provided complementary information but showed comparatively weaker associations with central VA. Visual function in daily life extends beyond central VA alone. Tasks such as reading and mobility rely heavily on parafoveal and perifoveal visual function, which may be better captured by broader EZ integrity metrics. Prior studies have shown that reading speed and functional performance decline disproportionately when parafoveal photoreceptors are compromised, even when central VA remains relatively preserved [21,22]. Panmacular EZ attenuation and volume metrics may be more relevant for functional domains that depend on parafoveal and perifoveal vision, rather than for central VA alone. Together, these findings suggest that the relationship between EZ integrity and visual outcomes is inherently scale dependent. Central subfield metrics appear most informative for visual acuity-based endpoints, whereas broader macular assessments may better characterize global photoreceptor burden. Accordingly, future studies incorporating microperimetry may provide complementary information regarding the functional impact of parafoveal photoreceptor abnormalities, particularly when evaluating panmacular EZ attenuation and volume metrics. This distinction is particularly relevant when selecting structural biomarkers for specific clinical or research outcomes and emphasizes the importance of matching the spatial definition of OCT-derived metrics to the functional endpoint of interest.

The relationship between EZ integrity and drusen burden provides additional insight into the structural pathways underlying visual dysfunction in dry AMD. In analyses of the overall cohort, both EZ integrity metrics and drusen volume demonstrated associations with VA in selected multivariable models, indicating that each captures aspects of disease-related structural change. However, when analyses were stratified by the presence or absence of central subfield atrophy, the association between drusen volume and VA was attenuated, whereas EZ integrity metrics remained consistently associated with visual outcomes across strata. This pattern suggests that apparent drusen-driven associations with visual function may be influenced by disease stage and RPE involvement, whereas EZ integrity reflects a more direct link to photoreceptor structure and function. Such a distribution-dependent effect is conceptually consistent with a Simpson’s paradox-like phenomenon, in which associations observed in pooled analyses may be influenced by underlying structural heterogeneity. In this context, drusen volume may primarily reflect upstream disease burden, while EZ integrity represents downstream photoreceptor disruption that is more closely coupled to visual performance. Importantly, these findings do not diminish the biological relevance of drusen in the pathogenesis of dry AMD. Rather, they highlight the complementary roles of different OCT-derived biomarkers, with drusen volume capturing aspects of disease activity and risk, and EZ integrity providing a structural correlate more directly related to functional impairment. Additional analyses incorporating ONL-RPE thickness further supported this interpretation. Although ONL-RPE thickness was associated with baseline VA in cross-sectional models, it was not associated with Year 2 visual outcomes when evaluated alongside total EZ attenuation. This finding suggests that EZ-based measures may more directly reflect functionally relevant photoreceptor disruption than ONL-based thickness measures.

Our findings are consistent with and extend prior work on EZ integrity in dry AMD. In our previous report, Yordi et al. suggested that reduced EZ thickness and increased EZ attenuation were closely correlated with baseline VA and subsequent VA decline in a longitudinal cohort of dry AMD eyes [14]. Bell et al. validated the reproducibility of quantitative EZ and sub-RPE compartmental features across readers, highlighting their robustness as structural biomarkers [18]. Other groups have similarly emphasized the prognostic significance of EZ disruption: Pfau et al. reported that EZ loss correlated strongly with reduced retinal sensitivity and preceded geographic atrophy progression [13], while Abraham et al. demonstrated that outer retinal integrity influenced therapeutic response in dry AMD [23]. Compared with these prior reports, the present study uniquely links EZ integrity metrics with the clinically meaningful ≥ 15-letter loss endpoint, providing supportive evidence that structural OCT measures can reflect functional outcomes recognized in clinical trials. Importantly, the present analysis further delineates how the prognostic relevance of EZ integrity varies by both spatial scale and temporal outcome.

Recent regulatory developments underscore the potential of photoreceptor integrity as a clinical trial endpoint in dry AMD. Regulatory discussions with the FDA have suggested that structural OCT-derived biomarkers, including EZ integrity, could be considered as exploratory endpoints in early-phase trials for dry AMD [24] and macular telangiectasia [25], while ≥15-letter loss remains the established functional threshold of clinical significance. In parallel, several functional tests have been investigated as potential endpoints in AMD clinical trials. Low-luminance VA has been shown to detect earlier functional impairment and predict future visual decline [26]. Microperimetry, including targeted approaches, provides topographic retinal sensitivity maps and has demonstrated strong correlations with photoreceptor integrity, including EZ preservation [27]. Dark adaptation testing has also been explored, particularly in earlier disease stages [28]. While these assessments offer valuable complementary information, VA and ≥15-letter loss remain the primary regulatory benchmarks. In this context, our findings provide supportive evidence that EZ integrity metrics may serve as structural correlates of this functional endpoint, reinforcing their potential validity as clinically meaningful biomarkers. Nevertheless, additional validation in multicenter cohorts and prospective studies will be needed to confirm these findings and evaluate their suitability for regulatory adoption.

This study has several limitations. First, its retrospective design limits control of confounding and introduces potential biases inherent to observational analyses. Second, the lack of standardized refractions may have introduced variability in VA measurements, potentially affecting the accuracy and comparability of functional outcomes. Third, the study was conducted at a single tertiary referral center using a single OCT device, which may limit generalizability. Fourth, the 2-year follow-up period may not fully capture the long-term natural history of dry AMD or the prognostic value of EZ integrity over longer timeframes. Fifth, while associations between EZ integrity and visual outcomes were robust, causal inferences cannot be made, and residual confounding by disease stage or unmeasured factors may persist despite multivariable adjustment. Additionally, eyes undergoing cataract surgery during follow-up were excluded to minimize confounding of longitudinal VA changes by surgery-related visual improvement. However, because patients requiring cataract surgery may differ systematically in age or disease severity, this exclusion may have introduced selection bias and may limit the generalizability of the longitudinal findings. In addition, although longitudinal changes in EZ integrity were evaluated, the availability of only baseline and Year 2 imaging precluded assessment of the temporal relationship between structural progression and visual decline. Finally, visual function was assessed using conventional VA alone. While VA is inherently subjective, OCT-based EZ integrity offers the advantage of providing an objective and standardized structural assessment. Additional functional measures, including microperimetry, low-luminance VA, and dark adaptation, were not evaluated and may provide complementary information regarding retinal function, particularly in eyes with extrafoveal disease. Accordingly, EZ integrity metrics should be interpreted as clinically relevant but incomplete structural correlates of visual function.

## 5. Conclusions

Measures reflecting central photoreceptor integrity, including EZ-RPE CST and central subfield EZ attenuation, showed particularly robust relationships with both baseline visual function and clinically meaningful vision loss at Year 2. Beyond central thickness alone, EZ attenuation metrics provided complementary information regarding broader macular photoreceptor disruption, supporting the concept that EZ integrity captures multiple spatial dimensions of disease-related retinal dysfunction. The consistent association of EZ metrics with visual outcomes, independent of drusen burden in stratified analyses, further highlights the relevance of photoreceptor integrity as a structural correlate of functional impairment. Together, these findings support the potential utility of EZ-based OCT biomarkers for disease characterization and prognostic assessment in dry AMD. By offering objective and reproducible measures of photoreceptor health, EZ integrity metrics may enhance patient stratification and inform the selection of clinically meaningful endpoints in future observational studies and interventional trials.

## Figures and Tables

**Table 1:** Baseline characteristics of the study cohorts.

Characteristics	Overall (N = 351)	Cohort		
		No Atrophy (N = 224)	Any Macular Atrophy (N = 127)	Central Subfield Atrophy (N = 97)
Demographics				
Age	80.0 (73.0–85.0)	78.5 (71.3–85.0)	83.0 (78.0–87.0)	83.0 (78.0–87.0)
Sex, female	202 (57.5)	129 (57.6)	73 (57.5)	55 (56.7)
Clinical characteristics				
Baseline VA, ETDRS letters	76.2 (64.1–81.2)	78.2 (69.9–83.0)	64.1 (50.1–75.2)	60.1 (41.2–68.5)
Baseline VA categories				
ETDRS ≥ 80	113 (32.2)	98 (43.8)	15 (11.8)	6 (6.2)
70 < ETDRS < 80	88 (25.1)	64 (28.6)	24 (18.9)	12 (12.4)
ETDRS ≤ 70	150 (42.7)	62 (27.7)	88 (69.3)	79 (81.4)

Values are presented as median (IQR) for continuous variables and n (%) for categorical variables. Atrophy was defined as a cumulative area of total RPE loss of at least 0.05 mm^2^ (250 μm in diameter, cRORA equivalent). Central subfield atrophy represents a subset of macular (6 × 6-mm) atrophy with at least a portion of the lesion involving the central 1 mm subfield. ETDRS = Early Treatment Diabetic Retinopathy Study; RPE = retinal pigment epithelium; VA = visual acuity.

**Table 2:** Cross-sectional associations between EZ integrity and baseline VA.

Metrics	Region	ETDRS ≥ 80 (N = 113)	70 < ETDRS < 80 (N = 88)	ETDRS ≤ 70 (N = 150)	*p* Value	Spearman ρ (*p*)
EZ-RPE thickness (µm)	Central subfield	35.8 (30.9–38.5)	32.0 (20.6–36.4)	10.9 (0.01–30.1)	<0.001	0.59 (<0.001)
	Central macular	33.9 (30.1–35.7)	30.4 (22.4–34.0)	15.7 (1.43–31.0)	<0.001	0.56 (<0.001)
Partial EZ attenuation (%)	Central subfield	0.35 (0.00–8.35)	9.52 (0.02–41.7)	71.7 (6.94–100.0)	<0.001	−0.58 (<0.001)
	Central macular	1.09 (0.01–7.63)	8.36 (0.79–38.4)	64.4 (6.67–99.4)	<0.001	−0.55 (<0.001)
	Panmacular	0.37 (0.37–1.48)	2.48 (0.20–8.09)	11.6 (1.59–35.5)	<0.001	−0.53 (<0.001)
Total EZ attenuation (%)	Central subfield	0.00 (0.00–0.00)	0.00 (0.00–7.73)	36.7 (0.00–99.7)	<0.001	−0.60 (<0.001)
	Central macular	0.00 (0.00–0.19)	0.18 (0.00–8.84)	26.7 (0.00–85.4)	<0.001	−0.57 (<0.001)
	Panmacular	0.00 (0.00–0.10)	0.15 (0.00–1.60)	4.18 (0.18–21.8)	<0.001	−0.51 (<0.001)
EZ-RPE volume (mm^3^)	Panmacular	1.19 (1.12–1.25)	1.15 (1.06–1.21)	1.01 (0.75–1.17)	<0.001	0.48 (<0.001)
ONL-RPE thickness (µm)	Central subfield	156.5 (144.1–166.5)	151.6 (130.3–162.6)	116.0 (56.6–152.8)	<0.001	0.52 (<0.001)
	Central macular	139.1 (128.3–149.1)	132.3 (118.1–143.6)	102.0 (52.8–135.4)	<0.001	0.52 (<0.001)
Total RPE loss (%)	Central subfield	0.000 (0.000–0.000)	0.000 (0.000–0.000)	3.47 (0.000–59.3)	<0.001	−0.58 (<0.001)
	Central macular	0.000 (0.000–0.000)	0.000 (0.000–0.331)	4.76 (0.000–59.3)	<0.001	−0.56 (<0.001)
Drusen volume (mm^3^)	Central subfield	0.004 (0.001–0.010)	0.006 (0.002–0.017)	0.003 (0.001–0.009)	0.007	0.12 (0.020)
	Central macular	0.015 (0.007–0.038)	0.020 (0.007–0.058)	0.010 (0.003–0.028)	0.003	0.14 (0.008)

Values are presented as median (IQR) for continuous variables. *p* values were calculated using Kruskal–Wallis tests across VA categories and Spearman correlation with ETDRS letters. Central subfield indicates the 1 mm diameter region; central macular indicates the 2 mm diameter region; panmacular refers to the full 6 × 6 mm scan area. ETDRS = Early Treatment Diabetic Retinopathy Study; EZ = ellipsoid zone; ONL = outer nuclear layer; RPE = retinal pigment epithelium; VA = visual acuity.

**Table 3:** Cross-sectional multivariable analysis illustrating differential contributions of EZ integrity and drusen volume to baseline VA.

Cohort	EZ Metrics, 1 mm	EZ Metrics B (95% CI)	*p* Value	Drusen Volume, 1 mm (per 0.1 mm^3^) B (95% CI)	*p* Value
Overall (N = 351)	EZ-RPE CST (µm)	0.70 (0.59–0.82)	<0.001	21.7 (8.59–34.9)	0.001
	Partial EZ attenuation (%)	−0.26 (−0.30–−0.22)	<0.001	23.9 (10.8–37.0)	<0.001
	Total EZ attenuation (%)	−0.30 (−0.34–−0.26)	<0.001	11.1 (−1.33–23.4)	0.080
No central subfield atrophy (N = 254)	EZ-RPE CST (µm)	0.17 (0.02–0.33)	0.030	−3.58 (−13.7–6.52)	0.486
	Partial EZ attenuation (%)	−0.17 (−0.26–−0.09)	<0.001	0.38 (−9.20–9.96)	0.918
	Total EZ attenuation (%)	−0.10 (−0.15–−0.04)	0.001	0.55 (−9.88–11.0)	0.918
Central subfield atrophy present (N = 97)	EZ-RPE CST (µm)	0.90 (0.21–1.59)	0.011	65.4 (−20.1–150.9)	0.132
	Partial EZ attenuation (%)	−0.26 (−0.49–−0.02)	0.037	68.4 (−18.0–154.8)	0.119
	Total EZ attenuation (%)	−0.30 (−0.46–−0.14)	<0.001	57.9 (−25.1–140.9)	0.169

B (95% CI) and *p* values were estimated using multivariable linear regression models adjusted for age, sex, drusen volume (per 0.1 mm^3^), and each EZ metric. Central subfield atrophy was defined as the total RPE loss within the central 1mm subfield. B = unstandardized regression coefficient; CST = central subfield thickness; EZ = ellipsoid zone; RPE = retinal pigment epithelium; VA = visual acuity.

**Table 4:** Longitudinal associations of baseline EZ integrity with VA outcomes at Year 2.

Metrics	Region	Stable VA (<15 Letters Loss, n = 150)	Significant VA Loss (≥15 Letters Loss, n = 38)	*p* Value	Spearman ρ (*p*)
EZ-RPE thickness (µm)	Central subfield	31.5 (15.9–36.5)	7.43 (1.80–24.0)	<0.001	0.71 (<0.001)
	Central macular	30.4 (17.2–34.3)	13.6 (1.86–25.1)	<0.001	0.69 (<0.001)
Partial EZ attenuation (%)	Central subfield	7.18 (0.00–61.2)	82.7 (34.9–100.0)	<0.001	−0.71 (<0.001)
	Central macular	6.84 (0.33–51.9)	65.6 (22.5–99.6)	<0.001	−0.70 (<0.001)
	Panmacular	1.38 (0.11–11.4)	10.8 (4.19–46.8)	<0.001	−0.68 (<0.001)
Total EZ attenuation (%)	Central subfield	0.00 (0.00–23.4)	47.8 (1.20–76.5)	<0.001	−0.69 (<0.001)
	Central macular	0.04 (0.00–3.92)	29.4 (1.45–79.8)	<0.001	−0.68 (<0.001)
	Panmacular	0.05 (0.00–3.92)	2.95 (0.23–27.4)	<0.001	−0.68 (<0.001)
EZ-RPE volume (mm^3^)	Panmacular	1.15 (1.01–1.22)	1.04 (0.67–1.14)	<0.001	0.60 (<0.001)
ONL-RPE thickness (µm)	Central subfield	148.6 (122.5–161.4)	113.5 (93.9–140.2)	<0.001	0.67 (<0.001)
	Central macular	132.9 (103.2–143.8)	99.2 (69.6–123.2)	<0.001	0.66 (<0.001)
Total RPE loss (%)	Central subfield	0.00 (0.00–0.08)	1.13 (0.00–41.0)	0.003	−0.65 (<0.001)
	Central macular	0.00 (0.00–5.02)	5.82 (0.00–43.3)	0.007	−0.63 (<0.001)
Drusen volume (mm^3^)	Central subfield	0.004 (0.001–0.010)	0.006 (0.002–0.013)	0.302	0.08 (0.303)
	Central macular	0.015 (0.005–0.013)	0.022 (0.006–0.032)	0.362	0.11 (0.145)

Values are presented as median (IQR) for continuous variables. *p* values were calculated using Mann–Whitney U tests for between-group comparisons and Spearman correlation with VA at Year 2. Central subfield indicated the 1 mm diameter region; central macular indicated the 2 mm diameter region; panmacular referred to the full 6 × 6 mm scan area. EZ = ellipsoid zone; ONL = outer nuclear layer; RPE = retinal pigment epithelium; VA = visual acuity.

**Table 5:** Predictive and independent associations of baseline EZ integrity with Year 2 VA outcomes.

**Panel A.** Multivariable logistic regression for ≥ 15-letter loss						
**Models**	**OCT metrics** **OR** (**95%CI**)	**OCT metrics** ***p* value**	**AUC** (**95% CI**)	**Comparison**	**ΔAUC** (**95% CI**)	**DeLong** ***p* Value**
M1 (Age + Sex)	N/A	N/A	0.66 (0.56–0.75)	N/A	N/A	N/A
M2 (M1 + EZ-RPE CST (µm))	0.95 (0.92–0.97)	<0.001	0.76 (0.69–0.84)	M1 vs. M2	0.10 (0.01–0.20)	0.025
M3 (M1 + Partial EZ attenuation, 1 mm (%))	1.02 (1.01–1.03)	<0.001	0.77 (0.69–0.84)	M1 vs. M3	0.11 (0.02–0.20)	0.015
M4 (M1 + Total EZ attenuation, 1 mm (%))	1.01 (1.00–1.02)	0.005	0.73 (0.65–0.81)	M1 vs. M4	0.07 (0.00–0.15)	0.060
M5 (M1 + ONL-RPE thickness, 1 mm (µm))	0.99 (0.99–1.00)	0.070	0.69 (0.60–0.77)	M1 vs. M5	0.03 (−0.01–0.08)	0.163
M6 (M1 + Drusen volume, 1-mm (0.1 mm^3^))	2.19 (0.10–48.9)	0.620	0.66 (0.56–0.75)	M1 vs. M6	0.00 (−0.02–0.02)	0.842
**Panel B.** ANCOVA models for Year 2 VA						
**Metrics**	**EZ metrics** **B** (**95% CI**)		***p* value**	**Drusen volume** **1 mm** (**per 0.1 mm^3^**) **B** (**95% CI**)		***p* value**
EZ-RPE CST (µm)	0.68 (0.48–0.87)		<0.001	11.3 (−9.11–31.8)		0.275
Partial EZ attenuation, 1 mm (%)	−0.24 (−0.31–−0.18)		<0.001	14.9 (−5.58–35.4)		0.153
Total EZ attenuation, 1 mm (%)	−0.25 (−0.32–−0.17)		<0.001	3.03 (−17.5–23.5)		0.771

**Panel A.** ORs (95% CI) and *p* values were estimated using multivariable logistic regression models. AUC and ΔAUC were reported with 95% CI; *p* values for AUC comparisons were calculated using the DeLong test. Drusen volume was scaled per 0.1 mm^3^ for interpretability. **Panel B.** B (95% CI) and *p* values were estimated using ANCOVA models evaluating the association between EZ metrics and Year 2 VA, adjusted for baseline VA, age, sex, and drusen volume (per 0.1 mm^3^). B = unstandardized regression coefficient; CST = central subfield thickness; EZ = ellipsoid zone; ONL = outer nuclear layer; RPE = retinal pigment epithelium; VA = visual acuity.

## Data Availability

The data presented in this study are available from the corresponding author upon reasonable request due to privacy and ethical restrictions.

## Supplementary Materials

The following supporting information can be downloaded at: https://www.mdpi.com/article/10.3390/diagnostics16182925/s1, Table S1. Baseline characteristics of eyes included in and excluded from the longitudinal cohort; Table S2: Longitudinal changes in EZ integrity stratified by VA loss at Year 2; Table S3: Cross-sectional and longitudinal multivariable models including total EZ attenuation and ONL-RPE thickness.

## Abbreviations

The following abbreviations are used in this manuscript:

AMD	Age-related macular degeneration
BM	Bruch’s membrane
cRORA	Complete retinal pigment epithelium and outer retinal atrophy
CST	Central subfield thickness
ETDRS	Early Treatment Diabetic Retinopathy Study
EZ	Ellipsoid zone
FDA	The U.S. Food and Drug Administration
GA	Geographic atrophy
iAMD	Intermediate AMD
nAMD	Neovascular AMD
OCT	Optical coherence tomography
RPE	Retinal pigment epithelium
SD-OCT	Spectral-domain OCT
VA	Visual acuity
VEGF	Vascular endothelial growth factor
