# Supplementary material for: The Structure–Function Link of Ellipsoid Zone Integrity and Outer Retinal Features with Visual Acuity in Dry AMD
Source: Diagnostics (Basel). 2026 Sep 10;16(18):2925. doi: 10.3390/diagnostics16182925 (PMC13605958; doi:10.3390/diagnostics16182925)
Supplement: Supplementary file 1 [file diagnostics-16-02925-s001.zip › diagnostics-4466901-supplementary.pdf]

Table S1. Baseline Characteristics of Eyes Included in and Excluded from the Longitudinal Cohort.

Characteristics	Included (n=188)	Excluded (n=163)	P value
**Demographics**			
**Age**	78.5 (71.3–85.0)	80.0 (73.0–85.0)	0.322
**Sex, female n (%)**	107 (57.0)	95 (58.3)	0.796
**Clinical characteristics**			
**Baseline BCVA, ETDRS letters**	76.2 (64.1–82.0)	75.2 (63.1–80.2)	0.267
**EZ-RPE CST (μm)**	28.1 (7.21–35.9)	31.7 (11.6–36.9)	0.174
**Partial EZ attenuation, 1-mm (%)**	13.8 (0.07–85.0)	7.16 (0.00–71.6)	0.156
**Total EZ attenuation, 1-mm (%)**	0.39 (0.00–50.2)	0.00 (0.00–38.6)	0.223
**ONL-RPE thickness, 1-mm (μm)**	145.3 (113.3–158.6)	146.9 (117.7–164.1)	0.243
**Drusen volume, 1-mm (mm^3)**	0.004 (0.001–0.011)	0.004 (0.001–0.012)	0.494
**Central subfield atrophy presence, n (%)**	57 (30.3)	40 (24.5)	0.227

Values are presented as median (IQR) or n (%). Baseline characteristics were compared between eyes included in and excluded from the longitudinal cohort to assess potential selection bias. Continuous variables were compared using the Mann–Whitney U test and categorical variables using the χ^2 test.

BCVA = best corrected visual acuity; ETDRS = Early Treatment Diabetic Retinopathy Study; EZ = ellipsoid zone; ONL = outer nuclear layer; RPE = retinal pigment epithelium.

Table S2. Longitudinal Changes in EZ Integrity Stratified by VA Loss at Year 2.

Metrics	Region	Stable VA (< 15 letters loss, n = 150)	Significant VA loss (≥ 15 letters loss, n = 38)	P value
**Δ EZ-RPE thickness (μm)**	Central subfield	-1.70 (-4.77– 0.25)	-3.68 (-9.17– -0.22)	0.019
**Δ Partial EZ attenuation (%)**	Central subfield	0.85 (0.00–10.53)	4.85 (0.00–19.60)	0.161
	Central macular	3.04 (0.00–13.64)	6.85 (0.04–23.73)	0.082
**Δ Total EZ attenuation (%)**	Central subfield	0.00 (0.00–3.49)	11.32 (0.00–38.41)	< 0.001
	Central macular	0.10 (0.00–6.10)	14.77 (1.40–26.33)	< 0.001

Values were presented as median (IQR). Δ values represented changes from baseline to Year 2. Central subfield indicated the 1-mm diameter region; central macular indicated the 2-mm diameter region. P values were calculated using Mann–Whitney U tests for comparisons between eyes with and without significant visual acuity loss (≥ 15 letters).

EZ = ellipsoid zone; RPE = retinal pigment epithelium; VA = visual acuity.

Table S3. Cross-sectional and Longitudinal Multivariable Models Including Total EZ Attenuation and ONL-RPE Thickness.

Panel A. Linear regression model for baseline BCVA

**Metrics**	**EZ metrics B (95% CI)**	**P value**	**ONL-RPE thickness, 1-mm (μm) B (95% CI)**	**P value**
**Total EZ attenuation, 1-mm (%)**	-0.17 (-0.25– -0.09)	< 0.001	0.11 (0.05–0.18)	< 0.001

Panel B. ANCOVA model for Year 2 BCVA

**Metrics**	**EZ metrics B (95% CI)**	**P value**	**ONL-RPE thickness, 1-mm (μm) B (95% CI)**	**P value**
**Total EZ attenuation, 1-mm (%)**	-0.28 (-0.40– -0.16)	< 0.001	-0.04 (-0.13–0.06)	0.455

Panel A. B (95% CI) and P values were estimated using a multivariable linear regression model adjusted for age, sex, Total EZ attenuation, drusen volume (per 0.1 mm^3), and ONL-RPE thickness.

Panel B. B (95% CI) and P values were estimated using an ANCOVA model evaluating the association between Total EZ attenuation and Year 2 BCVA, adjusted for baseline BCVA, age, sex, drusen volume (per 0.1 mm^3), and ONL-RPE thickness.

B = unstandardized regression coefficient; BCVA = best corrected visual acuity; EZ = ellipsoid zone; ONL = outer nuclear layer; RPE = retinal pigment epithelium.
